# Whole-genome sequence of the Cooley spruce gall adelgid, *Adelges cooleyi* (Hemiptera: Sternorrhyncha: Adelgidae)

**DOI:** 10.1093/g3journal/jkad224

**Published:** 2023-09-28

**Authors:** Dustin T Dial, Kathryn M Weglarz, Bryan M T Brunet, Nathan P Havill, Carol D von Dohlen, Gaelen R Burke

**Affiliations:** Department of Entomology, University of Georgia, Athens, GA 30602, USA; Department of Biology, Utah State University, Logan, UT 84322, USA; Ottawa Research and Development Centre, Agriculture and Agri-Food Canada, Ottawa, ON, Canada K1A 0C6; USDA Forest Service, Northern Research Station, Hamden, CT 06514, USA; Department of Biology, Utah State University, Logan, UT 84322, USA; Department of Entomology, University of Georgia, Athens, GA 30602, USA

**Keywords:** *Adelges cooleyi*, adelgid, Adelgidae, phylloxera, aphid, Aphidomorpha, genome assembly, mitochondria

## Abstract

The adelgids (Adelgidae) are a small family of sap-feeding insects, which, together with true aphids (Aphididae) and phylloxerans (Phylloxeridae), make up the infraorder Aphidomorpha. Some adelgid species are highly destructive to forest ecosystems such as *Adelges tsugae*, *Adelges piceae*, *Adelges laricis*, *Pineus pini*, and *Pineus boerneri*. Despite this, there are no high-quality genomic resources for adelgids, hindering advanced genomic analyses within Adelgidae and among Aphidomorpha. Here, we used PacBio continuous long-read and Illumina RNA-sequencing to construct a high-quality draft genome assembly for the Cooley spruce gall adelgid, *Adelges cooleyi* (Gillette), a gall-forming species endemic to North America. The assembled genome is 270.2 Mb in total size and has scaffold and contig N50 statistics of 14.87 and 7.18 Mb, respectively. There are 24,967 predicted coding sequences, and the assembly completeness is estimated at 98.1 and 99.6% with core BUSCO gene sets of Arthropoda and Hemiptera, respectively. Phylogenomic analysis using the *A. cooleyi* genome, 3 publicly available adelgid transcriptomes, 4 phylloxera transcriptomes, the *Daktulosphaira vitifoliae* (grape phylloxera) genome, 4 aphid genomes, and 2 outgroup coccoid genomes fully resolves adelgids and phylloxerans as sister taxa. The mitochondrial genome is 24 kb, among the largest in insects sampled to date, with 39.4% composed of noncoding regions. This genome assembly is currently the only genome-scale, annotated assembly for adelgids and will be a valuable resource for understanding the ecology and evolution of Aphidomorpha.

## Introduction

The Adelgidae, Phylloxeridae, and Aphididae comprise Aphidomorpha, a clade of sap-feeding insects that are cyclically parthenogenetic and exhibit complex, multigenerational, and polyphenic life cycles (Havill and Foottit 2007). The highly host-specific Adelgidae and Phylloxeridae are comparatively species poor, containing about 60–70 species each, whereas Aphididae contains approximately 5,000 species (http://aphid.speciesfile.org/). This disparity is likely an outcome of an ancient switch from gymnosperm to angiosperm feeding within Aphididae, and the subsequent rapid radiation of angiosperms (von Dohlen and Moran 2000). In contrast, adelgids retained the comparatively species-poor gymnosperm conifers as hosts, and phylloxerids exploited only a few angiosperm families. Some adelgid species are invasive pests of major economic and ecological importance, such as the hemlock woolly adelgid (*Adelges tsugae*) (Reardon *et al.* 2011; Havill *et al.* 2016) and the balsam woolly adelgid (*Adelges piceae*) (Amman 1965; Havill *et al.* 2021). While several high-quality aphid genomes and one phylloxeran genome have been published, there are currently no adelgid genomes, limiting the phylogenetic scope of genomic resources within Aphidomorpha.

The phylogenetic reconstruction of relationships within Adelgidae and among Aphidomorpha has been complicated by complex polyphenism, a lack of informative morphological characteristics, and a lack of genomic data. The deep divergences among Adelgidae, Phylloxeridae, and Aphididae likely date to the Jurassic (Havill *et al.* 2007). Adelgids and phylloxerans are distinguished from Aphididae by a lack of cornicles, by reproduction by means of ovipary in every generation, and by a lack of *Buchnera*, the ancestral nutritional endosymbiont of aphids. While obligate nutritional symbioses are absent in the phylloxera, adelgids exhibit a diverse mosaic of symbionts wherein each major host lineage contains a unique pair (von Dohlen *et al.* 2017). While some studies have suggested that aphids and phylloxerans are sister groups on the basis of morphological characters such as reduced ovipositors (Grimaldi and Engel 2005), most studies support adelgids + phylloxerans as sister to the aphids with fossil data (Heie and Wegierek 2009), mitochondrial and/or ribosomal sequences (von Dohlen and Moran 1995; Havill *et al.* 2007; Yeh *et al.* 2020), or phylogenomic datasets (Rispe *et al.* 2020; Hardy *et al.* 2022; Owen and Miller 2022). However, these studies did not specifically address the relationship among adelgids, phylloxerans, and aphids, and often contained few taxa or little genomic data within Adelgidae.

**Fig. 1.**
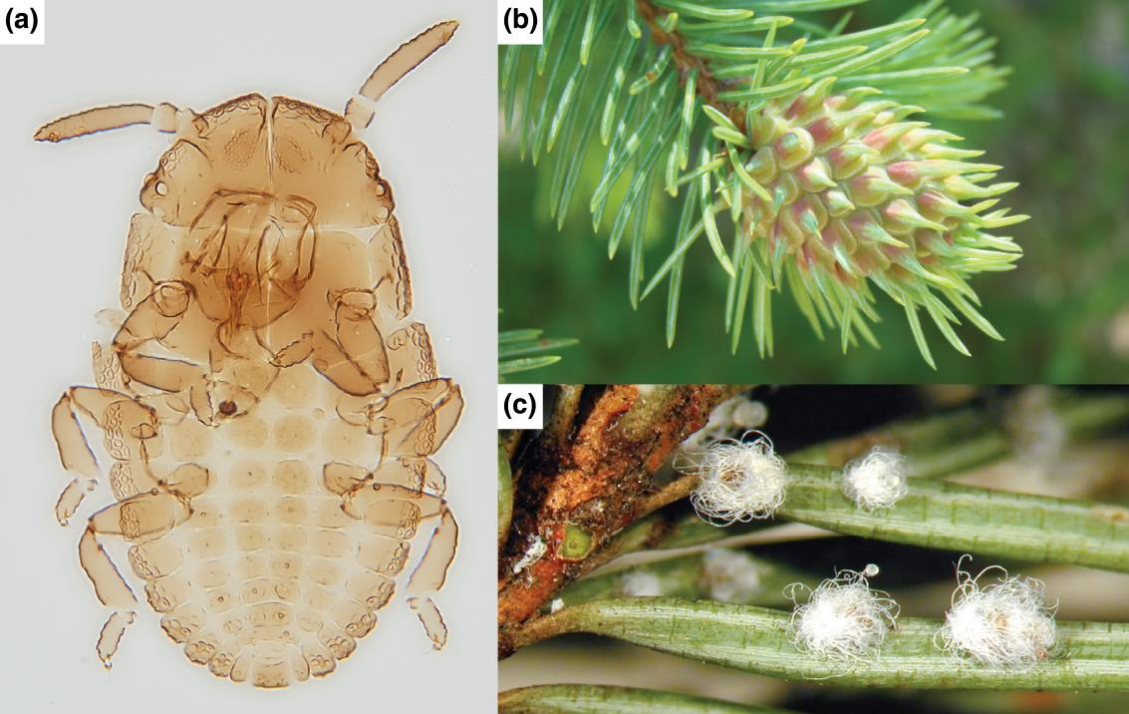
Ecology of *A. cooleyi*. a) Slide-mounted 1st instar *A. cooleyi* (image credit: N. Havill). b) *A. cooleyi* gall (image credit: D. Dickinson). c) *A. cooleyi* on Douglas fir (image credit: M. Montgomery).

*Adelges cooleyi* (Gillette), the Cooley spruce gall adelgid, is native to western North America and is distributed throughout the Rocky Mountains and Cascade Mountains (Fig. 1a; Gillette 1907). An analysis of 2 mitochondrial genes and amplified fragment length polymorphisms (AFLPs) indicated that there are at least 3 mitochondrial lineages within *A. cooleyi*: southeastern Arizona, Pacific Northwest, and Rocky Mountains (Ahern *et al.* 2009). AFLPs suggest that populations from Arizona are genetically isolated from the other 2 lineages. *Adelges cooleyi* feeds on spruce (*Picea*) species and Douglas fir (*Pseudotsuga menziesii*). On spruce, the insect induces the formation of large galls at the growing tips of branches and feeds on parenchyma tissue within the gall (Fig. 1b). On Douglas fir, it feeds on the phloem sap of needles, discoloring the needles and causing cosmetic damage (Fig. 1c; Annand 1928). Both trees are required to complete its 2-year life cycle, although cases of populations residing exclusively on spruce have been documented (Annand 1928; Cumming 1962). On spruce, the insects reproduce both sexually and asexually over the course of several generations, and winged asexuals emerge from galls to fly to Douglas fir in the summer (Annand 1928). On Douglas fir, they reproduce asexually, and winged generations return to spruce the following summer, or wingless asexuals can continue to reproduce on Douglas fir. As with the vast majority of other sap-feeding insects (Moran 2001), the plant sap diet of adelgids is supplemented with essential amino acids and vitamins by obligate intracellular bacteria residing within specialized tissues called bacteriomes. The alternation between different dietary sources (i.e. phloem and parenchyma) has been hypothesized to have promoted the multiple replacements of endosymbionts within Adelgidae (von Dohlen *et al.* 2017; Weglarz *et al.* 2018; Dial *et al.* 2022).

Here, we report a high-quality genome sequence of *A. cooleyi*, the first available genome representing Adelgidae. We include both nuclear and mitochondrial genomes. Our *A. cooleyi* genome data were generated alongside our efforts to recover the endosymbiont genomes (Dial *et al.* 2022). We report on the characteristics of both genomes and perform a phylogenomic analysis to test the relationships within Aphidomorpha. These publicly available genome data will provide a resource to help address future questions relating to adelgid systematics and evolution and to facilitate comparative analyses with the insects of other groups.

## Methods

### Insect collection, DNA extraction, and sequencing

Adelgids used for sequencing were collected from Franklin County, ID, USA, on state highway ID-36 in Emigration Canyon in July 2019 and identified by C.D.v.D. Galls containing parthenogenetic females were collected from blue spruce (*Picea coloradensis*) and cut open to reveal insect galleries. Approximately 100 individual whole-body 4th instar individuals were collected from the gall, and DNA was extracted using a DNeasy Blood & Tissue Kit (Qiagen Inc., Germantown, MD, USA). Adelgids within a gall are likely all clonal offspring of a single fundatrix (Havill and Foottit 2007), but intergall migration is possible and could result in occupants belonging to different clones (Ozaki 1995). Size selection was performed by using the BluePippin system (Sage Science, Beverly, MA, USA), yielding DNA fragments of an average size of 29,055 bp. DNA was sequenced using the Pacific Biosciences (PacBio) (Menlo Park, CA, USA) Sequel II System in continuous long-read mode on a single SMRTCell at the Georgia Genomics and Bioinformatics Core.

### Transcriptome sequencing

To aid in ab initio gene prediction, RNA sequencing was performed on a total of 4 whole-body *A. cooleyi* samples collected from both spruce (gallicolae) and Douglas fir (sistens). Total RNA was extracted from whole bodies with the Zymo Research DirectZol RNA miniprep kit. Quantification and quality assessment were performed by using an Agilent 2100 Bioanalyzer. Strand-specific libraries were prepared using the Illumina TruSeq Stranded mRNA Sample Prep kit with oligo dT selection and were sequenced with the HiSeq system with 125 bp paired-end chemistry at the University of Utah Huntsman Cancer Institute.

### Genome assembly, contaminant filtering, and annotation

**Fig. 2.**
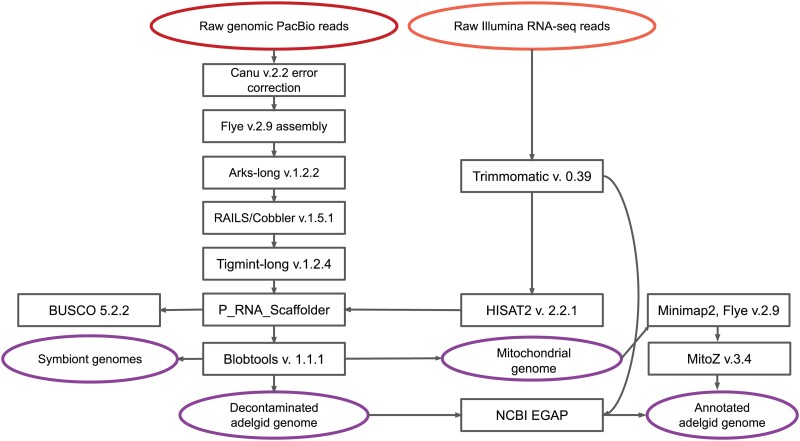
Summary schematic of tools used for genome assembly and annotation.

Genome assembly was performed in a multistep process (Fig. 2). First, consensus reads were generated from overlapping raw PacBio reads by correction with Canu v. 2.2 (Koren *et al.* 2017). The Canu-corrected reads were then assembled with Flye v. 2.9 (Kolmogorov *et al.* 2019) with the option –pacbio-corr (<3% error) and with 5 rounds of genome polishing. Scaffolding was initially performed with ARKS-long (Coombe *et al.* 2018), with options “m = 8-10000c = 4l = 4 a = 0.3 k = 20 j = 0.05.” Rails and Cobbler (Warren 2016) were then used for further scaffolding and gap closing with minimap2 (Li 2018) (options “250 0.8 250 bp 2 pacbio”). The resulting assembly was corrected with Tigmint-long (Jackman *et al.* 2018) with options “span = auto G = 272898873 dist = auto longmap = pb.” Corrected reads were used in all scaffolding and gap-closing steps. Finally, P_RNA_Scaffolder (Zhu *et al.* 2018) was used to scaffold the genome with quality-trimmed RNA-seq reads aligned to the genome with HISAT2 (Kim *et al.* 2019). Settings were adjusted such that the minimal number of RNA-seq pairs to join contigs was 5 instead of the default value of 2 for additional stringency.

**Fig. 3.**
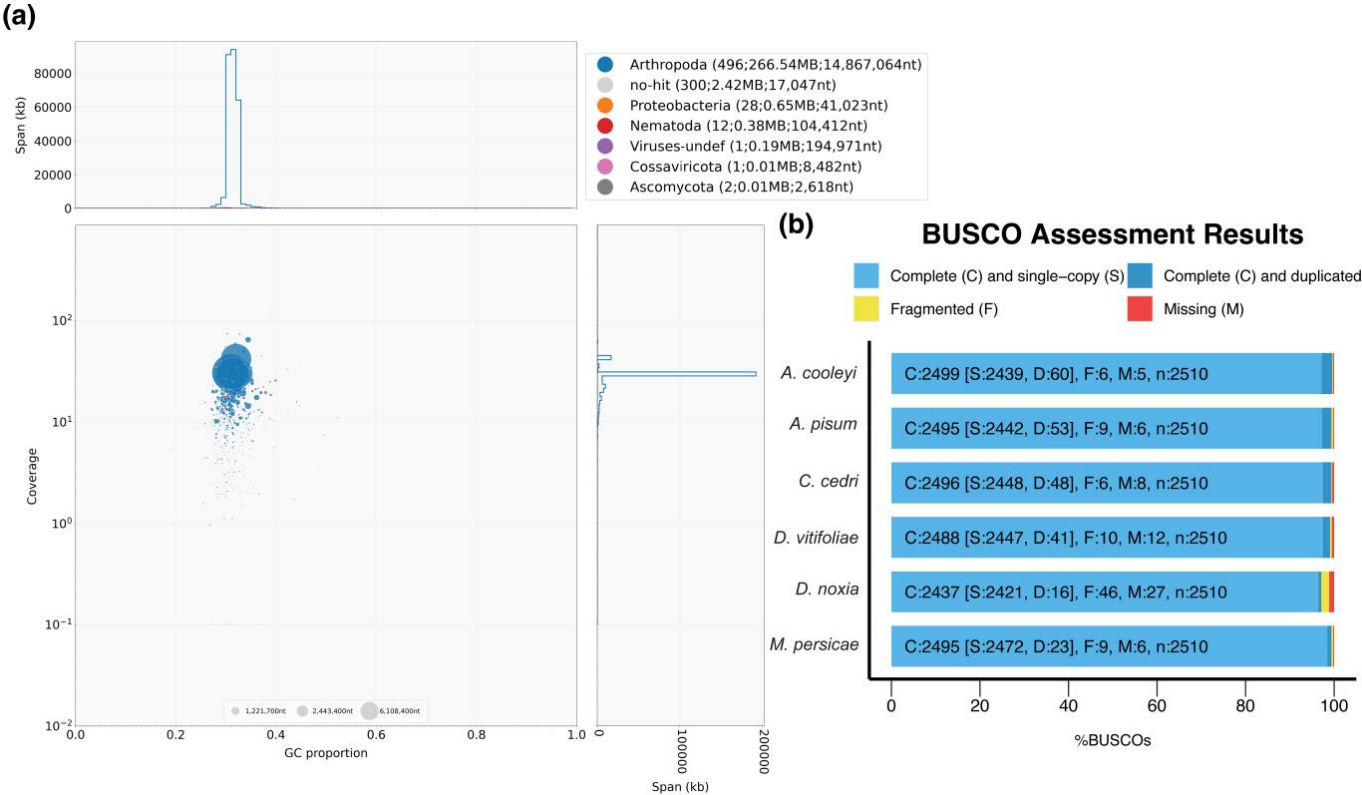
Quality assessment of the *A. cooleyi* genome assembly. a) A taxon-annotated GC-proportion and coverage plot of the *A. cooleyi* genome. Each blob represents a scaffold and is scaled by length and colored by taxonomy assigned by BlobTools with a custom RefSeq database. The *X* axis is the average GC content of each scaffold, and the *Y* axis corresponds to the read coverage based on the alignment of corrected PacBio reads. Most scaffolds assigned as noninsect taxa were revealed to belong to the insect genome upon searching their ORFs against NCBI's NR database. Contigs assigned to the “no-hit” category were not discarded. b) A comparison of the BUSCO results of *A. cooleyi* and several published high-quality aphid genomes using the Hemiptera gene set of 2,510 conserved single-copy genes.

Contaminants were screened and filtered with BlobTools (Laetsch and Blaxter 2017); corrected PacBio reads were aligned to the genome with minimap2, and open reading frames (ORFs) ≥100 bp were called using EMBOSS (Rice *et al.* 2000) and searched with DIAMOND v. 2.0.2 (Buchfink *et al.* 2021) against the RefSeq (Pruitt *et al.* 2007) database modified to include only arthropods, bacteria, archaea, viruses, nematodes, and fungi with TaxonKit (Shen and Ren 2021) for taxonomic assignment. The plots illustrating coverage, GC content, and taxonomy were generated with BlobTools and used to remove all contigs that were not hemipteran (Fig. 3a). DIAMOND search results of contigs assigned to noninsect groups (i.e. Proteobacteria, Nematoda, Ascomycota, and viruses) were manually checked and searched against NR before exclusion. In total, only 3 symbiont sequences (2 chromosomes and a plasmid), 1 6 kb contig belonging to Burkholderia, the mitochondrial genome, and contigs <200 bp were removed. BUSCO v. 5.3.2 (Manni *et al.* 2021) was used to assess genome quality and completeness, with MetaEuk (Levy Karin *et al.* 2020) used for gene prediction by default (Hemiptera_odb10 database, *n* = 2,510). Contigs with no hit (300 sequences) were retained as they may contain lineage-specific genes. After decontamination, assembly statistics were determined using the stats.sh script from BBmap version 38.98 (Bushnell 2014).

The resulting insect genome was annotated with the NCBI Eukaryotic Genome Annotation Pipeline (EGAP). This pipeline utilized our *A. cooleyi* RNA-seq data for gene prediction, in addition to protein alignments of NCBI RefSeq proteins from *Myzus persicae*, *Diuraphis noxia*, *Halyomorpha halys*, *Acyrthosiphon pisum*, *Bemisia tabaci*, *Drosophila melanogaster*, *Cimex lectularius*, 118,571 Insecta GenBank proteins, and 9,034 Insecta RefSeq proteins. RNA-seq and protein alignments were performed with STAR (Dobin *et al.* 2013) and ProSplign (https://www.ncbi.nlm.nih.gov/sutils/static/prosplign/prosplign.html), respectively, and were used for gene model prediction with *GNOMON*, the NCBI eukaryotic gene prediction tool (Souvorov *et al.* 2010). Repeats were masked with WindowMasker (Morgulis *et al.* 2006), and transcript and protein alignments were performed on the repeat-masked genome. The transcriptome and proteome generated by this pipeline were checked for completeness with BUSCO v4.1.4. The annotation release for *A. cooleyi* can be viewed online at https://www.ncbi.nlm.nih.gov/genome/annotation_euk/Adelges_cooleyi/100/ (GenBank assembly accession GCA_023614345.1).

### Mitogenome assembly and annotation

Our assembly screen revealed a noncircular contig with exceptionally high coverage and low GC content (50,776 bp and 14.6% GC). This contig was searched against the NCBI nt database (https://blast.ncbi.nlm.nih.gov/Blast.cgi) with BLASTn (Altschul *et al.* 1990) and matched to the *A. tsugae* mitochondrial genome (Yeh *et al.* 2020). Further inspection revealed that this contig contained 2 complete copies of the mitochondrial genome, approximately 24.2 and 26.6 kb in length, differing in the length of their control and repeat regions (4.2–5.6 and 5.2–6.2 kb, respectively). Corrected reads were mapped to this contig with minimap2 (515 reads) and reassembled with Flye v 2.9 in Geneious Prime 2021.1.1 (https://www.geneious.com) with default settings, yielding a circular 24 kb contig with 206-fold coverage. The genic content of this contig is nearly identical to that of the mitogenome copies present in the original 50.8 kb scaffold. To ensure that we excluded nuclear mitochondrial reads from the mitochondrial genome assembly, we mapped the mitochondrial reads back to the clean nuclear assembly with minimap2, and no hits were obtained. Furthermore, we employed a strategy that involved mapping all reads to the nuclear genome, extracting and mapping unmapped reads to the mitochondrial genome, and reassembling the reads that were mapped. This produced a 23.9 kb contig highly similar to the 24 kb contig derived earlier. Mitogenome annotations were produced using MitoZ v.3.4 (Meng *et al.* 2019) and compared with the *A. tsugae* mitogenome. The trnE gene was not annotated by MitoZ, but a comparison of this region with *A. tsugae* and related aphid *trnE* genes showed high-sequence similarity and was manually annotated. Tandem Repeat Finder was used to identify tandem repeats in the noncoding regions of the mitochondrial genome (Benson 1999).

### Incorporation of publicly available RNA-seq data

To increase taxon representation within Adelgidae and Phylloxeridae in our species tree, we included 3 adelgid and 4 phylloxeran species that each have an available transcriptome. Adelgid species included *A. tsugae* (SRR1198669), *Pineus* sp. pispAD (SRR5134714), and *Adelges* sp. AdspAS (SRR5134707). While the latter 2 taxa were not identified to the species level prior to sequencing, the extraction of full-length COI genes from assembled transcripts showed 99.85 and 100% sequence identity to *Pineus strobi* and *Adelges abietis*, respectively. Phylloxeran species included *Phylloxerina nyssae* (SRR23289299), *Phylloxera* sp. PhspAP35 (SRR5134737), *Phylloxera* sp. PE11a (SRR23290230), and *Phylloxera* sp. H7a (SRR23290233). Raw RNA-seq reads for these species were retrieved from the Sequence Read Archive (SRA) by using the SRA-toolkit v. 3.0.1, assembled with Trinity v. 2.8.5 (Grabherr *et al.* 2011), and set to trim the sequences automatically with Trimmomatic (Bolger *et al.* 2014). The longest isoform of each transcript was selected, and the prediction of proteins was performed by TransDecoder (https://github.com/TransDecoder/TransDecoder/) and set to output ORFs > 100 amino acids. The resulting ORFs were searched with BLASTp (*e*-value cutoff 1e^−5^) against a database of 18 species within the Aphidomorpha and 2 coccoid proteomes (Supplementary Table 1), and ORFs with no hits were removed with the TransDecoder Predict function. BUSCO v. 5.3.2 was used to assess the completeness of each quality-filtered transcriptome (Supplementary Table 2).

### Orthology inference and species phylogeny

We performed orthology clustering of predicted protein sequences from the *A. cooleyi* genome, the 3 adelgid transcriptomes, and 4 phylloxeran transcriptomes described above; from the genome sequences of *Daktulosphaira vitifoliae* (grape phylloxera) (Rispe *et al.* 2020), aphid species *Cinara cedri* (Julca *et al.* 2020), *Aphis glycines* (Mathers 2020), *A. pisum* (Li *et al.* 2019), and *M. persicae* (Mathers *et al.* 2017); and from 2 coccoid species, *Ericerus pela* (Yang *et al.* 2019) and *Phenacoccus solenopsis* (Li *et al.* 2020b) with OrthoFinder v2.5.2 (Emms and Kelly 2019). The longest isoform of each annotated gene was extracted and used as an input into OrthoFinder. We ran OrthoFinder in multiple sequence alignment mode (-M msa), and alignment and tree inference were performed within OrthoFinder with MAFFT (Katoh and Standley 2013) and FastTree (Price *et al.* 2010). This method estimates a species tree based on a concatenated alignment of all single-copy orthogroups present in all species. Alignments were lightly trimmed by OrthoFinder with default settings. To confirm the tree topology generated by OrthoFinder's utilization of FastTree, the concatenated alignment was used as input into IQ-Tree with automatic model selection with ModelFinder (JTT + F + R4) (Kalyaanamoorthy *et al.* 2017) and 1,000 ultrafast bootstraps. Additionally, we ran OrthoFinder in its default species tree inference mode, which uses STAG (Emms and Kelly 2018) for species tree inference. This method creates a consensus species tree from the most closely related genes within both single-copy and multicopy orthogroups. Trees were rooted by STRIDE (Emms and Kelly 2017) using gene duplication information.

## Results and discussion

### *Adelges cooleyi* genome characteristics

We generated 84.26 Gb (2,729,794 reads) of long-read PacBio data from a pool of clonal *A. cooleyi* individuals collected from a single gall and a total of 59.4 Gb of RNA-seq data from *A. cooleyi* sampled from spruce and Douglas fir hosts (Table 1). The error correction of PacBio reads with Canu generated 8.80 Gb of data (229,338 reads, N50/N90 54,907/42,252), and these reads were used to de novo assemble the *A. cooleyi* genome. The final assembled, scaffolded, corrected, and contaminant-filtered genome had an average coverage of 30.0× (calculated from corrected consensus reads), was 270.2 Mb in total length, and contained 962 contigs and 840 scaffolds (Table 2). The genomic GC content was low (31.4%) but comparable with other aphid genomes. The genome is highly contiguous, with contig and scaffold N50 statistics of 7.18 and 14.87 Mb, respectively. The *A. cooleyi* genome is contained within 2*n* = 22 chromosomes (Steffan 1968), and 90% or more of the current assembly is contained within 59 scaffolds (L90 = 59) (Table 2). The prefiltered assembly possessed a low degree of contamination; most of the contigs identified by BlobTools as potential contaminants were either symbiont sequences or contained insect genes as verified by BLAST searches against NR (Fig. 3a). These contigs containing insect genes were retained in the final assembly. Although not assembled to the level of chromosomes, this genome is highly complete, with an excellent representation of conserved hemipteran genes, which meets or exceeds that of published genomes of related species (Fig. 3b, Table 3). Our assembly pipeline utilized error-corrected consensus reads from high-coverage PacBio data and likely produced an assembly with a low error rate (Dohm *et al.* 2020). However, we note that the lack of high-accuracy short-read data inhibits us from obtaining higher-level quality metrics such as kmer spectra or Merqury QV statistics (Rhie *et al.* 2020). The 2 endosymbiont species *Ca.* Vallotia cooleyia and *Ca.* Gillettellia cooleyia were described previously with the same long-read dataset (Dial *et al.* 2022). WindowMasker repeat-masked 43.67% of the genome, which falls within the range of masked content in related insect genomes. Gene prediction with NCBI EGAP produced 24,967 coding sequences (CDSs) and 27,577 transcripts (Table 4).

**Table 1.** Summary statistics of *A. cooleyi* RNA-seq reads used for gene prediction.

Sequence Read Archive accession	Material	Read pairs	Number of bases (Gb)	Percent aligned reads
All	Aggregate of all aligned samples	237,817,810	59.4	92
SRR21113675	Whole-body 4th instar collected from Douglas fir	54,998,082	13.7	92
SRR21113676	Whole-body 4th instar collected from spruce	54,421,594	13.6	93
SRR21113677	Whole-body 4th instar collected from spruce	60,718,604	15.2	90
SRR21113678	Whole-body 4th instar collected from Douglas fir	67,679,530	16.9	92

**Table 2.** *Adelges cooleyi* genome assembly statistics.

Total assembly size (Mb)	270.2
Number of contigs	962
Contig N50 (Mb)	7.18
Contig L50	11
Contig L90	106
Max contig length (Mb)	21.44
Number of scaffolds	840
Scaffold N50 (Mb)	14.87
Scaffold L50	8
Scaffold L90	59
Max scaffold length (Mb)	24.43
Number of scaffolds >50 kb	194
Main genome in scaffolds >50 kb	96.62%

**Table 3.** Comparison of genome statistics between *A. cooleyi* and related species.

Species	Reference	Contig count (N50 Mb)	Scaffold count (N50 Mb)	Total length (Mb)	CDS count
*Adelges cooleyi*	This study	962 (7.18)	840 (14.87)	270.20	24,967
*Daktulosphaira vitifoliae*	GCF_025091365.1	17,226 (0.72)	8,544 (45.89)	282.59	29,510
*Cinara cedri*	Julca *et al.* (2020)	6,160 (0.28)	2,842 (1.23)	396.03	22,503
*Aphis glycines*	Mathers (2020)	1,024 (2.0)	941 (2.51)	303.15	19,750
*Rhopalosiphum maidis^a^*	Chen *et al.* (2019)	960 (9.05)	220 (93.3)	326.02	17,629
*Sitobion miscanthi^a^*	Jiang *et al.* (2019)	1,148 (2.05)	656 (36.26)	397.9	16,006
*Diuraphis noxia*	Nicholson *et al.* (2015)	49,379 (0.01)	5,641 (0.40)	393.0	19,097
*Myzus persicae^a^*	Mathers *et al.* (2021)	915 (4.17)	360 (69.5)	395.14	27,663
*Acyrthosiphon pisum^a^*	Mathers *et al.* (2021)	2,298 (0.53)	558 (126.6)	525.80	30,784

^*a*^Scaffolds have been assigned to chromosomes.

**Table 4.** Gene annotation summary statistics for *A. cooleyi*.

Feature	Count	Mean length (bp)	Median length (bp)	Min length (bp)	Max length (bp)
Genes	15,100	10,785	4,267	68	414,770
All transcripts	27,577	2,330	1,781	68	62,780
mRNA	24,967	2,447	1,871	96	62,780
misc_RNA	627	2,258	1,881	193	9,857
tRNA	212	74	73	71	84
lncRNA	1,613	1,050	758	83	34,541
rRNA	65	316	119	119	4,328
CDSs	24,967	1,778	1,275	96	61,812
Exons	128,259	271	171	1	34,139
Introns	110,635	1,695	189	30	404,309

### *Adelges cooleyi* has large mitochondrial control and repeat regions and a gene order identical to the ancestral Insecta

**Fig. 4.**
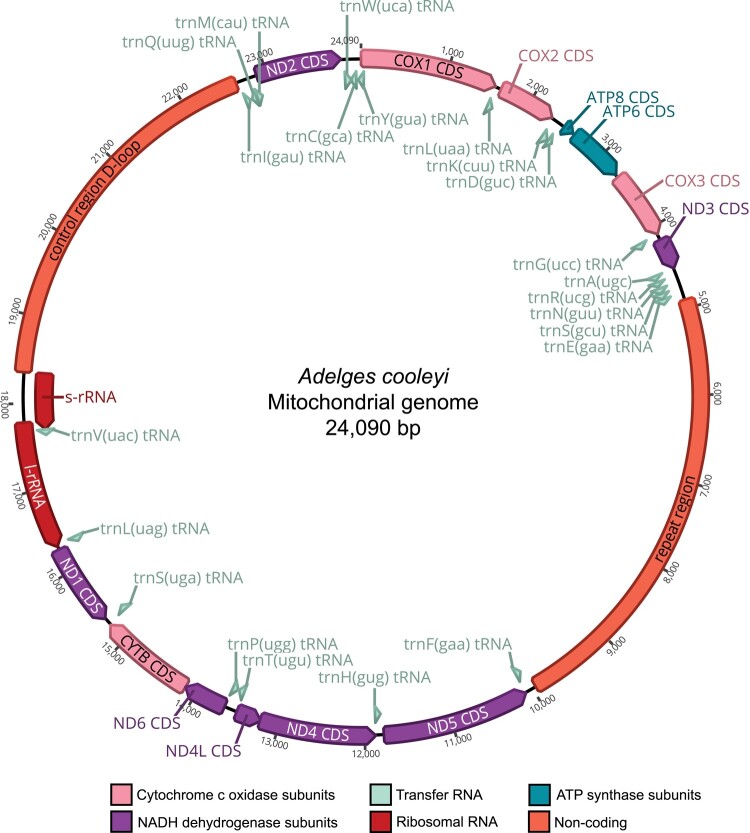
Circular mitochondrial genome map for *A. cooleyi*.

Our initial assembly yielded a 50.8 kb linear concatemer containing 2 copies of the mitochondrial genome, each with complete gene sets but differing in the length of the control and repeat regions. These noncoding regions share 98% nucleotide identity between copies but differ in length. Concatemers are common when read length exceeds the length of the sequence being assembled (Hunt *et al.* 2015), and the length variation of noncoding regions in the mitogenome copies may be attributed to the presence of different clones within the same gall or heteroplasmy within a single clone. Upon mapping corrected reads to this contig and reassembling these isolated mitochondrial reads, we obtained a final circular, 24,090 bp contiguous sequence with even 206.0× coverage and 85.2% AT content (Fig. 4). This genome size is consistent with the length of mitogenome sequences as seen in individual reads. The gene content and order are identical to the ancestral insect (Cameron 2014), with 13 protein-coding genes, 22 tRNA genes, and 2 rRNA genes, with 83.7% genic AT content. Notably, the *A. cooleyi* mitochondrial genome possesses a long 5.14 kb repeat region and a 4.31 kb control region with AT contents of 86.1 and 88.4%, respectively. Consequently, 39.4% of the mitochondrial genome is composed of noncoding DNA. These regions in the final 24 kb mitogenome are most similar to the first copy in the 50.8 kb contig. These noncoding regions in *A. cooleyi* are much longer than in the *A. tsugae* mitogenome, but the genic sequences of these assemblies share high sequence identity and the same gene order. While the overall size of the *A. cooleyi* mitogenome is significantly greater than the size of a typical insect mitogenome, it is not exceptionally large. For example, unusually large mitogenomes up to 36 kb have been described in weevils, up to 28 kb in Hercules beetles, and up to 26 kb in seed beetles due to large, often heteroplasmic control regions (Boyce *et al.* 1989; Sayadi *et al.* 2017; Morgan *et al.* 2022).

The control region is thought to function as a binding site for proteins involved in replication and transcription (Saito *et al.* 2005). Reconstructing the control region is challenging because of the presence of tandem repeat units that are often larger than the read length generated by short-read technologies. While often only partial or missing in public databases (Cameron 2014), it can vary in size from a few hundred to several thousand bases within the Hemiptera (Li and Liang 2018) and can be as long as 13.7 kb in the Coleoptera (Morgan *et al.* 2022). Within the Cicadidae, polymerase chain reaction-amplified control regions can range between 1.7 and 5 kb (Lukasik *et al.* 2019). The application of long-read sequencing to the study of mitogenomes has recently revealed large noncoding regions that were previously unassembled with short-read data in insects (Sayadi *et al.* 2017; Xu *et al.* 2018; Filipovic *et al.* 2021; Morgan *et al.* 2022) and other animals (Gan *et al.* 2019; Kinkar *et al.* 2020; Novosolov *et al.* 2022; Sharbrough *et al.* 2023). Our long-read data have allowed for the reconstruction of the complete control region in *A. cooleyi*, and while large, the size falls within the known range for related insects. Tandem Repeat Finder found 13 repeats in the control region, but the largest and highest scoring repeats occur in 3 stretches of 299 bp repeats with copy numbers of 3.5, 4.1, and 4.1 spanning a total of 3.5 kb.

Many aphid mitochondrial genomes exhibit a tandem repeat region typically located between trnE and trnF (Wang *et al.* 2013; Zhang *et al.* 2014, 2021, 2022; Wei *et al.* 2019). The size and tandem repeat copy number vary widely across different aphid lineages, ranging from a few hundred to a few thousand base pairs. The repeat region of *A. cooleyi* contains 61 tandem repeats, 59 of which are <30 bp and the 2 largest and highest scoring tandem repeat units are each composed of 9.0 and 11.1 copies of an identical 231 bp repeat spanning a total of 4.6 kb. This repeat matches the partially assembled repeat regions of *A. tsugae* (77.56% identity, 95% length) and the aphid species *Eutrichosiphum pasaniae* (71.23% identity, 91% length) (Li *et al.* 2020a) and *Cavariella salicicola* (77.27% identity, 34% length) (Wang *et al.* 2013) in BLASTn searches against NR. Recent studies have proposed that the repeat region either evolved in the common ancestor of aphids and was lost in several aphid lineages or evolved independently multiple times (Chen *et al.* 2019; Song *et al.* 2019). The presence of this region in *A. cooleyi* and *A. tsugae* supports the hypothesis that the repeat region is an ancestral feature of Aphidomorpha. The ancient origin of the repeat region and its persistence throughout their ∼245 My evolution (Johnson *et al.* 2018; Szwedo and Nel 2011) has raised questions about its origin and function. It has been proposed that the repeat region in aphids evolved from an ancient duplication of the control region and functions as a second origin of replication to enhance mitochondrial replication and transcription (Wang *et al.* 2015). Song *et al.* (2019) reported that the pairwise identity between the control and repeat regions for several aphid species is on average 38%. In *A. cooleyi*, these regions share 48.6% identity (2,199 identical sites). A second origin of replication may be related to the origin of cyclical parthenogenesis; a single female can produce hundreds of offspring over the course of her lifetime, a highly energy-demanding process (Song *et al.* 2019). Additionally, many host plants produce oxidizing molecules in defense against insects; increased rates of mitochondrial replication and recycling may be a mechanism by which aphids and adelgids deal with these host defenses.

### Phylogenomics supports Adelgidae and Phylloxeridae as sister groups

To investigate the phylogenetic position of *A. cooleyi* in relation to other adelgid species and to assess the position of Adelgidae relative to Phylloxeridae and Aphididae, we performed orthology clustering of protein CDS from 4 adelgid species, 5 phylloxera species, 4 aphid species, and 2 coccoid species. This sampling represents the 2 adelgid genera (*Adelges* and *Pineus*) and 4 of the 5 major adelgid lineages (those that feed on pine, hemlock, Douglas fir, and larch secondary hosts), 3 phylloxera genera (*Daktulosphaira*, *Phylloxerina*, and *Phylloxera*), 3 aphid tribes (Macrosphini, Aphidini, and Eulachnini), and the Coccidae and Pseudococcidae. OrthoFinder assigned 179,577 genes (91.6% of the total number of genes) to 21,768 orthogroups, with 3,583 orthogroups and 961 single-copy orthologs shared by all species. It should be noted that this does not necessarily represent a complete shared gene set, as transcriptome data were used for some species.

**Fig. 5.**
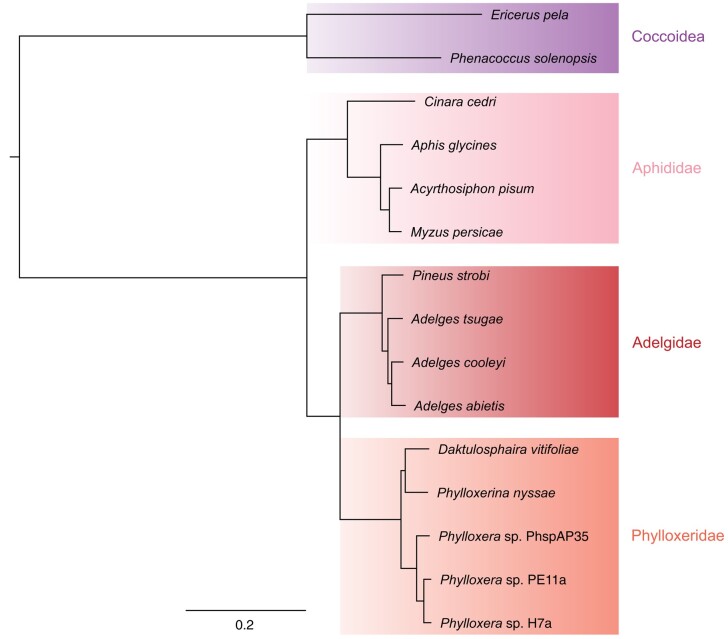
Maximum-likelihood phylogeny of selected aphid, adelgid, phylloxeran, and coccoid species with sequenced genomes and transcriptomes based on a concatenated alignment of 961 single-copy orthologs and 1,000 ultrafast bootstraps in IQ-tree. *Adelges tsugae*, *A. abietis* (*Adelges* sp. adspAD), *P. strobi* (*Pineus* sp. pispAD), *P. nyssae*, *Phylloxera* sp. PhspAP35, *Phylloxera* sp. PE11a, and *Phylloxera* sp. H7a gene sets originate from transcriptomic data, and the remaining taxa are from genomic datasets. Clades are colored by family, and branch lengths represent amino acid substitutions per site. The bootstrap support at all branches was 100%.

Maximum likelihood analysis with IQ-tree of the 961 concatenated single-copy orthologs generated a fully resolved species tree with 100% support at all nodes (Fig. 5). This topology is identical to the species trees produced by FastTree and STAG within OrthoFinder. Our species tree identifies phylloxerans as sister to adelgids and Adelgidae and Phylloxeridae as sister to Aphididae. The topology within Aphididae is consistent with previous analyses (von Dohlen *et al.* 2006; Novakova *et al.* 2013; Julca *et al.* 2020; Hardy *et al.* 2022; Owen and Miller 2022; Smith *et al.* 2022), and the topology within Adelgidae mirrors the relationships established using 3 mitochondrial genes and 1 nuclear gene (Havill *et al.* 2007).

Our phylogenomic results are consistent with the morphological and reproductive features that support the grouping of adelgids and phylloxerans to the exclusion of aphids. The possession of intimate symbioses with obligate nutritional bacteria is ubiquitous throughout the aphids and adelgids but absent in the phylloxerans and is thus most likely a plesiomorphic trait (Ponsen 1997). It has been hypothesized that the ancestral phylloxera lost their obligate symbionts and symbiotic cells known as bacteriocytes as a result of specialized feeding on modified parenchyma cells containing essential amino acids, relaxing selection on the maintenance of genes underlying symbiont nutritional pathways (Buchner 1965; Warwick and Hildebrandt 1966; von Dohlen *et al.* 2017). One intriguing question is the developmental and evolutionary origin of the cells that contain symbionts within Aphidomorpha. Parsimony would suggest that bacteriocytes were present in the common ancestor of the Aphidomorpha but were lost in the phylloxera and maintained within the adelgids and aphids. However, similar symbiotic cells have evolved independently in multiple insect orders (Buchner 1965) and even within families (Kuechler *et al.* 2019), suggesting that the true evolutionary scenario could be more complex. Alternatively, adelgids may have evolved their bacteriocytes independently from aphids, which, if true, may bear some influence on the comparative characteristics of symbiosis within the adelgids and aphids. Future work exploring these questions could reveal a more comprehensive picture of the evolution of symbiosis within this group.

## Conclusions

We have generated a high-quality draft genome of *A. cooleyi* using PacBio long-read data and high-coverage Illumina RNA-seq data. This genome is the first representative of the adelgid lineage and adds to a small but growing list of high-quality genomes within Aphidomorpha. As an outgroup of Aphididae, it will aid in the detailed comparative analysis of aphid biology and evolution. Furthermore, we anticipate that it will enhance the studies of insect–microbe interactions relating to adelgid bacterial endosymbionts and the history of symbioses within Aphidomorpha.

## Supplementary Material

jkad224_Supplementary_Data

## Data Availability

The nuclear and mitochondrial genome assemblies and annotations for this project are available under RefSeq accession GCF_023614345.1 and GenBank accession OQ991941.1, respectively. PacBio long-read sequences and RNA-seq reads are available under the BioProject accession numbers PRJNA827457 and PRJNA870591, respectively. The code used for the assembly pipeline is available on GitHub (https://github.com/dustin-dial/Adelges_cooleyi_genome). Supplemental material available at G3 online.
