# Supplementary material for: Whole-genome sequence of the Cooley spruce gall adelgid, Adelges cooleyi (Hemiptera: Sternorrhyncha: Adelgidae)
Source: G3 (Bethesda). 2023 Sep 28;14(1):jkad224. doi: 10.1093/g3journal/jkad224 (PMC10755206; doi:10.1093/g3journal/jkad224)
Supplement: jkad224_Supplementary_Data [file jkad224_supplementary_data.zip › Supplemental_Table_1_G3-2023-404302.docx]

Supplementary Table 1. Aphid and coccoid datasets used to identify ORFs from adelgid and phylloxeran transcriptomes. Only ORFs with hits to known proteins were retained for phylogenomic analysis. NCBI accessions and aphidBase versions are provided.

| **Genus** | **Species** | **Common name** | **Accession** |
| --- | --- | --- | --- |
| *Adelges* | *cooleyi* | Cooley spruce gall adelgid | GCA_023614345.1 |
| *Acyrthosiphon* | *pisum* | pea aphid | GCF_005508785.1 |
| *Sitobion* | *miscanthi* | Indian grain aphid | GCA_008086715.1 |
| *Diuraphis* | *noxia* | Russian wheat aphid | GCF_001186385.1 |
| *Myzus* | *cerasi* | black cherry aphid | aphidBase v1.1 |
| *Myzus* | *persicae* | green peach aphid | GCF_001856785.1 |
| *Pentalonia* | *nigronervosa* | banana aphid | GCA_014851325.1 |
| *Aphis* | *glycines* | soybean aphid | aphidBase v6.0_bt1 |
| *Aphis* | *gossypii* | cotton aphid | GCF_004010815.1 |
| *Rhopalosiphum* | *maidis* | corn leaf aphid | GCF_003676215.2 |
| *Chaitophorus* | *viminalis* | small black and green willow aphid | JAIPRK000000000 |
| *Stegophylla* | sp. | woolly oak aphid | JAIPRM000000000 |
| *Cinara* | *cedri* | pine bark aphid | GCA_902439185.1 |
| *Eriosoma* | *lanigerum* | woolly apple aphid | GCA_013282895.1 |
| *Geopemphigus* | sp. | pistacia gall aphid | JAIPRN000000000 |
| *Pemphigus* | *obesinymphae* | cottonwood petiole gall aphid | JAIPRL000000000 |
| *Hormaphis* | *cornu* | witch hazel aphid | GCA_017140985.1 |
| *Daktulosphaira* | *vitifoliae* | grapevine root-aphid | aphidBase v3.1 |
| *Ericerus* | *pela* | Chinese white wax scale insect | GCA_011428145.1 |
| *Phenacoccus* | *solenopsis* | cotton mealybug | GCA_009761765.1 |
