# Supplementary material for: Whole-genome sequence of the Cooley spruce gall adelgid, Adelges cooleyi (Hemiptera: Sternorrhyncha: Adelgidae)
Source: G3 (Bethesda). 2023 Sep 28;14(1):jkad224. doi: 10.1093/g3journal/jkad224 (PMC10755206; doi:10.1093/g3journal/jkad224)
Supplement: jkad224_Supplementary_Data [file jkad224_supplementary_data.zip › Supplemental_Table_2_G3-2023-404302.docx]

Supplementary Table 2. BUSCO scores (Hemiptera database) of quality-filtered transcriptomes included in the species tree.

| Species | *Adelges tsugae* | *Adelges* sp. adspAD | *Pineus* sp. pispAD | *Phylloxerina nyssae* | *Phylloxera* sp. PhspAP35 | *Phylloxera* sp. PE11a | *Phylloxera* sp. H7a |
| --- | --- | --- | --- | --- | --- | --- | --- |
| Accession | SRR1198669 | SRR5134707 | SRR5134714 | SRR23289299 | SRR5134737 | SRR23290230 | SRR23290233 |
| Total ORFs | 39,616 | 16,185 | 10,872 | 13,884 | 14,283 | 14,364 | 13,632 |
| Complete BUSCOs | 94.4% (2,366) | 87.4% (2,195) | 85.1% (2,136) | 87.1% (2,185) | 88.9% (2,231) | 89.6% (2,247) | 92.0% (2,311) |
| Complete and single copy BUSCOs | 90.8% (2,279) | 80.8% (2,029) | 82.2% (2,063) | 85.8% (2,153) | 86.7% (2,175) | 87.4% (2,193) | 90.2% (2,265) |
| Complete and duplicated BUSCOs | 3.5% (87) | 6.6% (166) | 2.9% (73) | 1.3% (32) | 2.2% (56) | 2.2% (54) | 1.8% (46) |
| Fragmented BUSCOs | 1.6% (39) | 4.3% (109) | 2.0% (49) | 4.8% (120) | 3.3% (82) | 2.7% (68) | 2.8% (71) |
| Missing BUSCOs | 4.1% (105) | 8.3% (206) | 12.9% (325) | 8.1% (205) | 7.8% (197) | 7.7% (195) | 5.2% (128) |
